# Automated Assessment of Ki-67 Labeling Index Using Cell-Level Detection and Classification in Whole-Slide Images

**DOI:** 10.3390/diagnostics16050816

**Published:** 2026-03-09

**Authors:** Masayuki Tsuneki, Meng Li, Fahdi Kanavati

**Affiliations:** Medmain Research, Medmain Inc., 2-4-5-104, Akasaka, Chuo-ku, Fukuoka 810-0042, Japan

**Keywords:** Ki-67, labeling index, pathology, AI, detection, classification

## Abstract

**Background**: The Ki-67 labeling index (LI) is a widely used marker of tumour proliferation, yet its manual assessment is time-consuming and subject to substantial inter-observer variability. Automated methods may improve reproducibility, but their clinical relevance depends on achieving performance comparable to expert pathologists. **Method**: We evaluated an artificial intelligence (AI)-based, cell-level system for automated Ki-67 LI assessment that detects and classifies individual tumour cell nuclei as Ki-67-positive or -negative. After nuclear detection using a pre-existing cell detection model, a lightweight convolutional neural network classifier operating on nucleus-centred patches was trained, and then applied to cases independently assessed by three pathologists. Agreement between AI-derived and human Ki-67 LI values was compared directly with inter-pathologist agreement across a range of proliferation levels. **Results**: The AI-based cell classification achieved 98% AUC on a test set consisting of 71K positive and 170K negative image patches centred on nuclei. On the automated Ki-67 LI assessment, the AI system demonstrated concordance with expert pathologists comparable to human inter-observer variability. **Conclusions**: These results support the potential of cell-level automated Ki-67 assessment as a reproducible decision-support tool for routine histopathological practice.

## 1. Introduction

Ki-67 is a nuclear protein expressed in proliferating cells and is widely used as an immunohistochemical marker of cellular proliferation in routine surgical pathology. The Ki-67 labeling index (LI), defined as the proportion of Ki-67-positive tumor nuclei relative to the total number of tumor nuclei, is routinely reported in multiple tumour types and contributes to tumour grading, prognostic stratification, and therapeutic decision-making. For example, Ki-67 evaluation provides important prognostic and predictive information and has become an integral component of routine histopathological reporting, particularly in hormone receptor-positive, HER2-negative breast carcinomas [1]. As a result, accurate and reproducible assessment of Ki-67 LI is of substantial clinical importance, as even modest differences in the reported value may influence risk stratification and treatment selection.

In current clinical practice, Ki-67 LI assessment is performed manually by pathologists, typically through visual estimation or manual counting in selected regions of interest [2,3]. Although this approach is common practice, it is inherently subjective and time-consuming [4,5]. Variability in hotspot selection, counting strategy, and interpretation of staining intensity can lead to differences in reported Ki-67 LI values, particularly in cases with intermediate or borderline proliferation indices where clinical decisions may be affected [3,6]. In addition, inter-laboratory differences in staining protocols, antibody clones, and slide preparation further contribute to measurement variability, complicating standardization efforts across institutions.

With the increasing adoption of whole-slide imaging in routine pathology, there is growing interest in automated methods that may improve the reproducibility and efficiency of Ki-67 assessment. Automated analysis offers the potential to process large tissue areas objectively and consistently, thereby reducing observer-dependent bias and workload. From a clinical perspective, however, the primary question is not whether an automated system introduces novel methodology, but whether it can perform at a level comparable to expert pathologists and operate within the range of established inter-observer variability. Any such system must therefore be evaluated relative to human performance rather than against an abstract technical benchmark, and its output should be interpretable within the framework of existing pathological practice.

Recent advances in computational pathology have enabled reliable detection and classification of individual cell nuclei on histopathological images using deep learning-based methods [7,8,9,10,11,12,13,14]. Cell-level analysis is particularly well suited to Ki-67 assessment [15], as the clinical definition of the labeling index is inherently based on counting individual positive and negative nuclei. By explicitly detecting and classifying individual cells, automated approaches have the potential to closely mirror the pathological assessment process while offering improved consistency, scalability, and traceability of results.

In this study, we evaluate an AI-based, cell-level approach for Ki-67 LI assessment that combines automated nuclear detection with classification of individual nuclei as Ki-67-positive or Ki-67-negative. The system was trained using a dataset of manually annotated cells and applied to a set of cases independently assessed by three pathologists.

Our primary objective was to determine whether the AI-derived Ki-67 labeling index demonstrates agreement with expert pathologists comparable to inter-pathologist agreement. We do not propose a new AI architecture; instead, we treat the AI system as an off-the-shelf tool and focus on a clinically grounded evaluation framework: a traceable, cell-level output (per-nucleus positivity) and benchmarking of AI-derived Ki-67 LI against a multi-pathologist reference with performance interpreted in relation to inter-observer agreement. This design directly addresses the practical question of whether an existing AI system performs within the variability expected in routine practice. By directly comparing AI performance to that of multiple pathologists and situating the results within the context of human inter-observer variability, this study aims to assess the potential clinical utility of automated Ki-67 LI assessment as a reproducible decision-support tool in routine histopathological practice. Such a system may contribute to improved standardization of Ki-67 evaluation [16,17,18], support quality assurance, and ultimately enhance the reliability of proliferation assessment in precision oncology.

## 2. Methods

### 2.1. Overview of the Analysis Pipeline

The objective of this study was to develop and evaluate an AI-based method for quantitative assessment of the Ki-67 LI on digitised histopathology images. To do so, an annotated dataset was first constructed to train the AI-based method.

**Figure 1:**
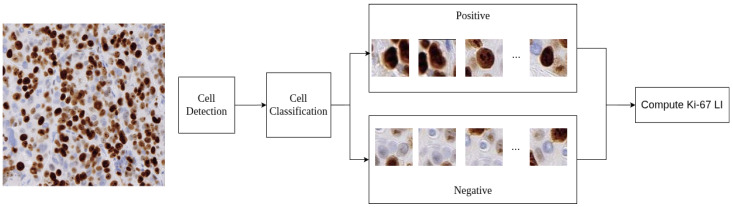
An overview of the AI-based Ki-67 labeling index computation. An image patch is passed through the cell detection stage followed by the cell classification into Ki-67-positive or Ki-67-negative. The Ki-67 LI is computed as a final step.

The AI-based method consists of two sequential steps: (1) automatic detection of individual cell nuclei using a deep learning-based instance segmentation algorithm, and (2) classification of detected nuclei as Ki-67-positive or Ki-67-negative, followed by computation of the Ki-67 LI. An overview of this is shown in Figure 1.

### 2.2. Clinical Cases

**Figure 2:**
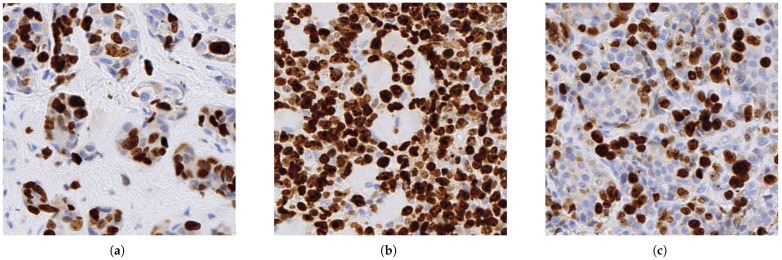
Representative samples of Ki67-positive cases in breast invasive ductal carcinoma. The samples are as follows: (**a**) scattering positive, (**b**) densely positive, and (**c**) intermediately positive.

For the present retrospective study, a total of 320 human biopsy and surgical cases of Ki-67 immunohistochemical stained histopathological specimens were collected from the surgical pathology files of International University of Health and Welfare, Mita Hospital (Tokyo) after histopathological review of those specimens by surgical pathologists. The 320 specimens consist of 169 breast cancers, 48 lung cancers, 37 stomach cancers, 35 colon cancers, and 31 uterine cancers. The Ki-67 antibody clone was MIB-1. For immunostaining, Dako automated staining was used. The tumour types across the cases were as follows: breast cancers: invasive ductal carcinoma; lung cancers: adenocarcinoma and squamous cell carcinoma; stomach cancers: adenocarcinoma; colon cancers: adenocarcinoma; and uterine cancers: endometrial carcinoma. Figure 2 shows representative samples from breast invasive ductal carcinoma.

The experimental protocol was approved by the ethical board of the International University of Health and Welfare (No. 19-Im-007). All research activities complied with relevant ethical regulations and were performed in accordance with relevant guidelines and regulations in the International University of Health and Welfare, Mita Hospital (Tokyo). Informed consent to use histopathological samples and pathological diagnostic reports for research purposes had previously been obtained from all patients prior to the surgical procedures and the opportunity for refusal to participate in research had been guaranteed by an opt-out manner.

### 2.3. Whole-Slide Image Preparation

All analyses were performed on digitised histopathology images acquired from immunohistochemically stained Ki-67 slides. Whole-slide images (WSIs) were scanned at high resolution using a Leica Aperio AT2 scanner and stored in a pyramidal format. All WSIs were scanned at a magnification of ×20.

### 2.4. Pathologist Reference Annotations

Regions of interest (ROIs) containing representative tumour tissue were selected by an experienced pathologist for annotation, making sure that an ROI does not include artefacts, non-tumour tissue, and large areas of background. The size of a given ROI image patch was 512 × 512 pixels.

**Figure 3:**
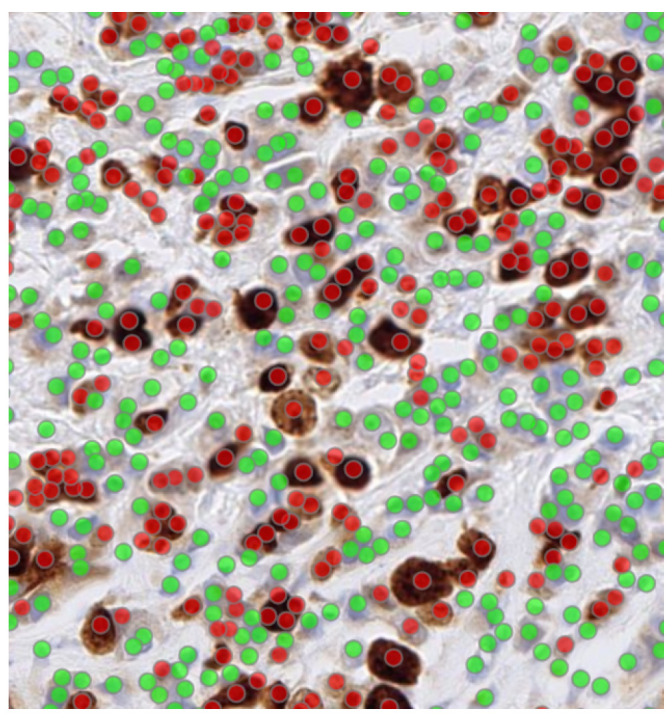
Annotation example showing Ki-67-positive (red) and Ki-67-negative (green).

The annotated dataset was constructed by expert manual annotation of individual nuclei on Ki-67-stained ROI images. Using an in-house annotation software, each nucleus was annotated as either Ki-67-positive or Ki-6-negative based on nuclear staining intensity and morphology, following standard pathological criteria (an example is shown in Figure 3). All stained nuclei were included, regardless of staining intensity; specifically, all tumour nuclei showing any degree of nuclear Ki-67 staining were labelled as positive (i.e., no intensity thresholding was applied). Annotations were reviewed by a senior pathologist to ensure consistency and to minimise ambiguous cases. From across the 320 cases, a total of 142K Ki-67-positive and 340K Ki-67-negative cells were annotated.

For evaluation of inter-observer variability and comparison with the AI-based method, a total of 20 unannotated ROI images were selected, each ROI from a different WSI. The Ki-67 LI estimates were then independently provided by three pathologists, each with more than ten years of experience. Each pathologist assessed the same set of image patches according to their individual routine diagnostic practice. An expert consensus value was defined as the median Ki-67 LI among the three pathologists and was used as the primary reference for comparison with the AI model.

### 2.5. Cell Detection

Detection of individual cell nuclei was performed using a fine-tuned StarDist algorithm [19,20], a deep learning-based instance segmentation method designed for star-convex object detection in microscopy images. StarDist models each nucleus as a star-convex polygon defined by radial distances from a central point, enabling accurate separation of densely packed and partially overlapping nuclei. The model was fine-tuned from the base model (Versatile H&E nuclei) using a subset of the training images following the same training protocol as described in [19,20]. We used the publicly available code [21] without any modifications.

A StarDist model was applied to the Ki-67-stained image patches to identify and segment individual nuclei. The output of this step consisted of polygonal nuclear masks and corresponding centroid coordinates for each detected cell. Only nuclei detected within tumour regions were retained for subsequent analysis.

### 2.6. Ki-67 Cell Classification

Following nuclear detection, each segmented nucleus, in patches of size 32 × 32 pixels, was classified as Ki-67-positive or Ki-67-negative. For this purpose, a supervised classification model consisting of a small convolutional neural network (CNN) was trained using a manually curated dataset.

The dataset was split at the case level into approximately 45% for training, 5% for validation, and 50% for testing. The reason for this split is as follows: classifying the cells into Ki-67-positive and Ki-67-negative is an extremely simple task as it is highly correlated with colour. In our initial experiments, even a simple classifier based on colour threshold was enough to achieve 91% AUC. We found that adding further training images in this particular case made no improvement in performance.

The classifier was implemented as a sequential CNN. Input patches were first rescaled to the [0, 1] range by dividing pixel intensities by 255. During training, data augmentation was applied to improve robustness to staining and orientation variation, including random horizontal/vertical flips, small rotations, and modest brightness/contrast jitter. The network then applied two convolutional blocks with 3 × 3 kernels (32 and 64 filters, respectively), each followed by max pooling. The resulting feature maps were flattened and passed through a 128-unit fully connected layer. A single sigmoid-activated output unit produced the probability of Ki-67 positivity (binary classification). Gelu activations were used in the hidden layers. The model was trained with the Adam optimiser, binary cross-entropy loss, and accuracy as the primary metric, for 20 epochs.

To address class imbalance, class weights were computed from the training set based on the inverse of class frequencies and supplied during optimisation. Model selection was performed using a checkpoint callback that saved the weights of the epoch with the lowest validation loss.

Image patches centred on detected nuclei were extracted and used as input to the classifier. The trained model was then applied to all detected nuclei to generate per-cell Ki-67 positivity predictions.

**Figure 4:**
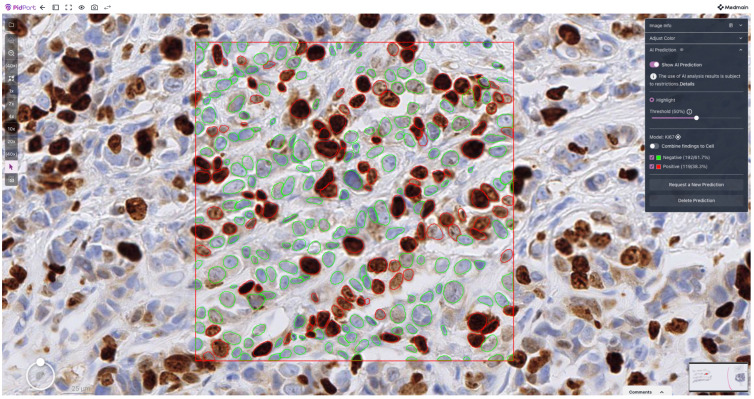
Example of cell detection and classification output on a selected area of a WSI.

Figure 4 shows representative final classification output on a selected area of a WSI.

### 2.7. Computation of the Ki-67 Labeling Index (LI)

The Ki-67 LI was computed as the ratio of Ki-67-positive nuclei to the total number of detected nuclei within a given region or case:

$$\text{Ki-67}\;\mathrm{LI}=\frac{N_{\text{Ki-67}+}}{N_{\mathrm{total}}}$$

For reporting purposes, the Ki-67 LI was expressed as a percentage. This definition is consistent with standard clinical and pathological practice.

### 2.8. Statistical Analysis

Correlation between AI predictions and pathologist estimates was quantified using Pearson’s correlation coefficient and Spearman’s rank correlation coefficient. Prediction error was assessed using the mean absolute error (MAE).

Bias and limits of agreement between AI predictions and expert consensus were evaluated using Bland–Altman analysis. For threshold-based analyses, clinically relevant Ki-67 cutoffs were applied and categorical agreement was assessed using Cohen’s κ statistic.

For the threshold-based analyses, Ki-67 LI values were grouped into three ordinal categories using commonly applied clinical cut-offs (<10%, 10–20%, and >20%). These thresholds were chosen to reflect typical low/intermediate/high proliferation strata similar to ranges used in some prior studies [22,23]. The optimal Ki-67 thresholds are not universal and may vary by tumour type, clinical context, and institutional practice; therefore, the cut-off analysis in this study is intended as a pragmatic sensitivity analysis rather than as a tumour-specific recommendation.

All statistical analyses were performed using Python 3.11-based scientific computing libraries. A two-sided significance level of 0.05 was used where applicable. The CNN classifier was implemented using Keras with TensorFlow backend [24].

## 3. Results

### 3.1. Classification

**Figure 5:**
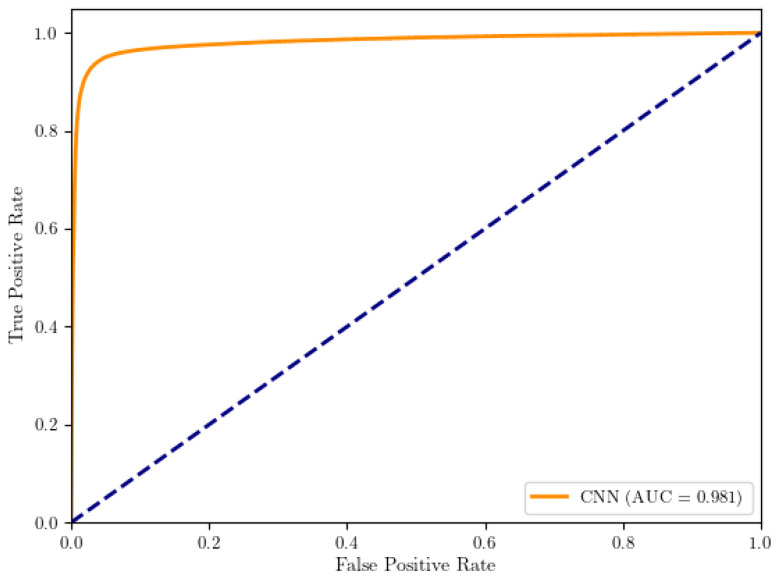
Cell classification ROC.

The cell classification model was evaluated on a test set consisting of 71K positive and 170K negative nuclei. These nuclei were sampled from held-out cases across the included tumour types (breast, lung, stomach, colon, and uterine cancers) to reflect multi-tumour variability. The receiver operator curve (ROC) is plotted in Figure 5.

### 3.2. Inter-Pathologist Agreement in Ki-67 Assessment

The primary aim of this analysis was to quantify inter-observer variability in Ki-67 LI assessment and to evaluate whether an AI-based approach achieves agreement comparable to that observed among expert pathologists. Agreement among the three pathologists was assessed using the intraclass correlation coefficient (ICC). The inter-pathologist ICC indicated good agreement, with an ICC of 0.93 (95% CI: 0.82–0.97).

**Figure 6:**
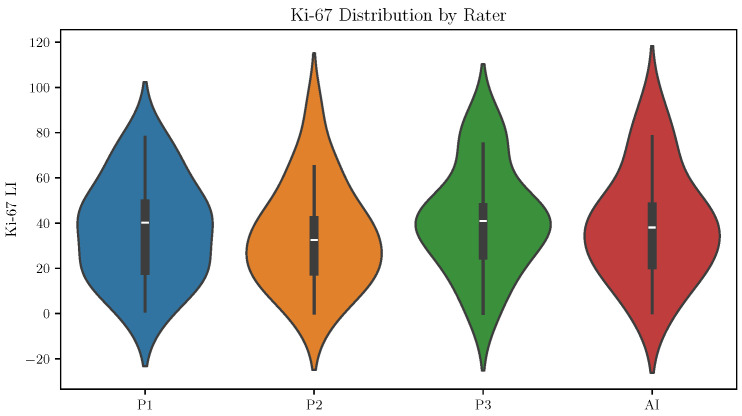
Distribution of Ki-67 labeling index across pathologists and AI predictions.

The distributions of Ki-67 LI values across pathologists were broadly similar, although small differences in dispersion were observed (Figure 6).

### 3.3. Agreement Between AI Predictions and Pathologists

Next, we evaluated the agreement between the AI model and individual pathologists, as well as with the expert consensus defined as the median Ki-67 LI among the three pathologists. Correlation analysis demonstrated a strong association between AI predictions and expert consensus (Pearson’s r=0.99, Spearman’s ρ=0.95), with a mean absolute error of 3.3 percentage points (Table 1).

When the AI model was included as an additional rater alongside the three pathologists, the overall agreement remained comparable to human-only agreement, with an ICC of 0.95 (95% CI: 0.89–0.98). This indicates that the variability between AI and pathologists falls within the range of inter-observer variability observed among experts.

**Figure 7:**
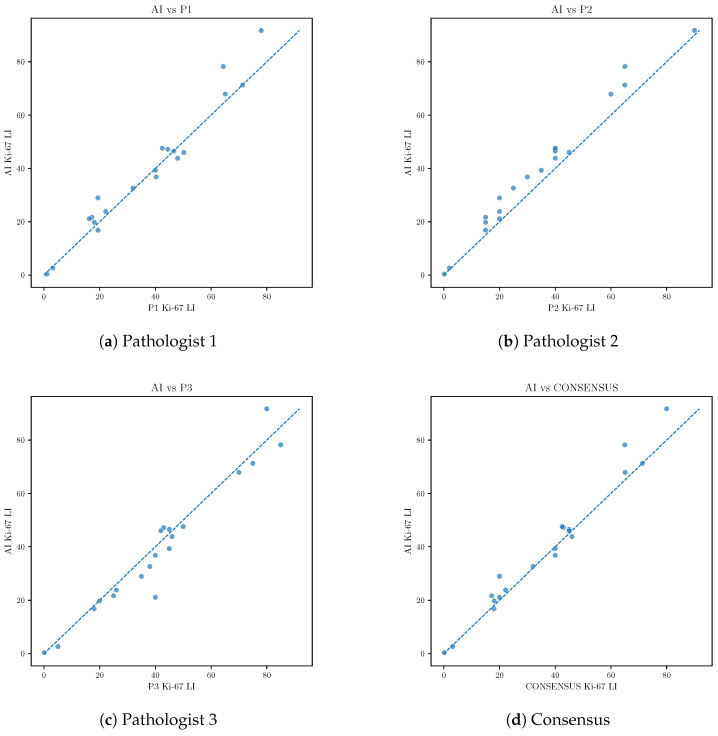
Scatter plots comparing AI-predicted Ki-67 labeling index with individual pathologists and expert consensus. The dashed line indicates identity.

Scatter plots comparing AI predictions with individual pathologists and expert consensus demonstrated close alignment along the identity line across the full dynamic range of Ki-67 values, with no evident systematic deviation (Figure 7).

### 3.4. Bias and Limits of Agreement Analysis

**Figure 8:**
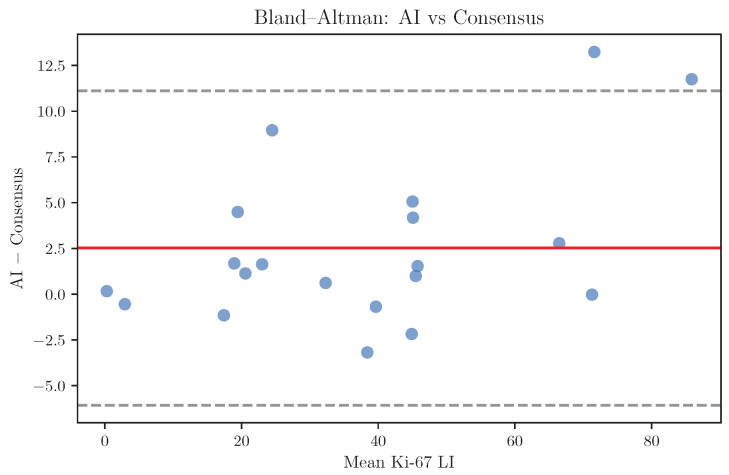
Bland–Altman analysis comparing AI-predicted Ki-67 labeling index to expert consensus. The solid line indicates mean bias; dashed lines indicate 95% limits of agreement.

Bland–Altman analysis was performed to further characterise systematic differences between AI predictions and expert consensus. The mean bias was 2.5 percentage points, indicating minimal systematic over- or under-estimation by the AI model (Figure 8).

No proportional bias was observed, and disagreement did not increase with higher Ki-67 LI values.

### 3.5. Threshold-Based Categorical Agreement

Using clinically relevant Ki-67 thresholds (<10%, 10–20%, >20%), categorical agreement between AI predictions and expert consensus was assessed. The resulting Cohen’s κ was 0.87, corresponding to substantial agreement. Table 2 shows a confusion matrix.

### 3.6. Summary of Findings

Taken together, these results demonstrate that the AI-based Ki-67 LI assessment achieves agreement with expert pathologists that is comparable to inter-pathologist agreement. The AI model exhibited low bias, strong correlation with expert consensus, and reduced variability relative to individual raters, supporting its potential utility as a reproducible decision-support tool within established clinical variability.

## 4. Discussion

In this study, we investigated whether an AI-based, cell-level approach to Ki-67 LI assessment can achieve performance comparable to that of expert pathologists. By explicitly positioning AI performance within the context of inter-pathologist variability, our results address a clinically meaningful question: whether automated Ki-67 assessment can reach a level of agreement consistent with routine pathological practice.

Inter-observer agreement among pathologists was good but imperfect, reflecting the variability inherent in manual Ki-67 assessment. These findings are consistent with previous reports highlighting the limitations of manual Ki-67 evaluation despite its widespread adoption and clinical importance [4,5,25,26].

The AI-based approach demonstrated strong agreement with individual pathologists as well as with expert consensus, with agreement metrics comparable to those observed among pathologists themselves. Notably, when the AI system was included as an additional rater alongside the three pathologists, the overall agreement did not deteriorate. This indicates that the variability between the AI system and human observers lies within the range of human–human variability. From a clinical perspective, this is a crucial observation, as it suggests that the AI system performs at a level consistent with expert interpretation rather than introducing systematic or clinically meaningful divergence.

Bias analysis further showed minimal systematic over- or underestimation of Ki-67 LI by the AI model relative to expert consensus. The limits of agreement between the AI and consensus were comparable to those observed between individual pathologists, supporting the interpretation that most discrepancies reflect the intrinsic uncertainty of Ki-67 assessment rather than algorithmic error.

Beyond agreement metrics, a key advantage of the proposed cell-level framework is its interpretability. Because the Ki-67 LI is derived from explicit detection and classification of individual nuclei, the outputs of the AI system can be directly inspected and verified by pathologists. This transparency distinguishes cell-based approaches from purely region-level or black-box regression models and is likely to facilitate clinical acceptance. Users can visually confirm which nuclei were classified as positive or negative, which is particularly valuable in cases with disagreement, where reviewing individual cell classifications may clarify the source of disagreement and support informed human oversight.

Another practical strength of the proposed method is its scalability and reproducibility. Once trained and validated, the AI system can analyse large WSIs in a consistent manner, without fatigue. This capability is especially advantageous in high-volume diagnostic laboratories, multicentre clinical trials, and large retrospective studies, where manual Ki-67 quantification is both time-consuming and subject to operator variability. Moreover, automated analysis enables more comprehensive assessment of tumour areas, potentially reducing sampling bias arising from limited hotspot selection and improving the representativeness of the measured LI.

Standardisation of Ki-67 assessment remains a long-standing challenge in pathology, despite extensive international efforts [27]. While AI alone cannot address all sources of variability, such as differences in staining protocols or pre-analytical factors, it can provide a stable and reproducible computational layer once slides are digitised. In this context, AI-based quantification may function as a harmonising tool that complements existing standardisation initiatives, helping to reduce observer-dependent variability and facilitating comparison of Ki-67 results across institutions and studies.

Furthermore, access to continuous and reproducible Ki-67 measurements may enable more nuanced use of proliferation indices in both research and clinical practice. Rather than relying solely on rigid categorical thresholds, AI-derived Ki-67 values could support probabilistic risk stratification models or be integrated with other quantitative histopathological and molecular features. Such integration is consistent with the broader direction of computational pathology and data-driven precision oncology.

It is also important to emphasise that the objective of this work is not to replace expert judgement, but to augment it. By providing an objective and reproducible reference, AI-based Ki-67 assessment may help reduce intra- and inter-observer variability, support the training of less experienced pathologists, and increase confidence in difficult or borderline cases. In this sense, the system should be viewed as a tool that strengthens the reliability of pathological evaluation while preserving the central role of the pathologist in diagnostic interpretation and clinical decision-making.

Several limitations of this study should be acknowledged. First, the classification model was trained on data obtained from a single source, which may limit generalisability. Second, only three pathologists participated in this study, all with a similar number of years of experience, which does not capture the full variability in experience between pathologists. Finally, the evaluation was retrospective and conducted on a defined dataset; further prospective validation and assessment across multiple institutions and tumour types will be necessary before routine clinical deployment can be considered.

From a clinical workflow perspective, the primary potential utility of this approach lies in its role as a decision-support tool rather than as a replacement for pathologists. Automated and reproducible Ki-67 quantification may help reduce variability, improve consistency across observers and institutions, and increase efficiency in high-volume settings. In particular, such a system may be especially valuable for cases with borderline Ki-67 values or in environments where access to subspecialty expertise is limited.

## 5. Conclusions

In conclusion, this study demonstrates that an AI-based, cell-level approach for Ki-67 LI assessment achieves agreement with expert pathologists comparable to inter-pathologist agreement. The AI system exhibited low bias, strong correlation with expert consensus, and variability consistent with routine clinical practice. These findings support the potential clinical utility of automated Ki-67 LI assessment as a reproducible and interpretable decision-support tool in histopathological workflows. Further prospective and multi-institutional studies will be required to confirm generalisability and to define the optimal role of such systems in clinical practice.

## Figures and Tables

**Table 1:** Agreement metrics comparing AI predictions to individual pathologists and expert consensus.

Comparison	Pearson *r*	Spearman ρ	MAE
AI vs. P1	0.98	0.95	3.87
AI vs. P2	0.99	0.98	5.10
AI vs. P3	0.97	0.96	4.37
AI vs. Consensus	0.99	0.95	3.30

**Table 2:** Agreement between AI and pathologists for Ki-67 labeling index displayed as a confusion matrix.

AI\Consensus	Low	Intermediate	High
Low	2	0	0
Intermediate	0	2	1
High	0	0	15

## Data Availability

The datasets generated during and/or analyzed during the current study are not publicly available due to specific institutional requirements governing privacy protection, but are available from the corresponding author upon reasonable request. The datasets that support the findings of this study are available from the International University of Health and Welfare, Mita Hospital (Tokyo, Japan), but restrictions apply to the availability of these data, which were used under a data use agreement that was made according to the Ethical Guidelines for Medical and Health Research Involving Human Subjects as set by the Japanese Ministry of Health, Labour and Welfare, and so are not publicly available. However, the data are available from the authors upon reasonable request for private viewing and with permission from the corresponding medical institution within the term of the data use agreement and if compliant with the ethical and legal requirements as stipulated by the Japanese Ministry of Health, Labour and Welfare.

## References

[1] Ma Q., Liu Y.B., She T., Liu X.L. (2024). The role of Ki-67 in HR+/HER2-breast cancer: A real-world study of 956 patients. Breast Cancer Targets Ther..

[2] Dowsett M., Nielsen T.O., A’Hern R., Bartlett J., Coombes R.C., Cuzick J., Ellis M., Henry N.L., Hugh J.C., Lively T. (2011). Assessment of Ki67 in breast cancer: Recommendations from the International Ki67 in Breast Cancer working group. JNCI J. Natl. Cancer Inst..

[3] Abubakar M., Orr N., Daley F., Coulson P., Ali H.R., Blows F., Benitez J., Milne R., Brenner H., Stegmaier C. (2016). Prognostic value of automated KI67 scoring in breast cancer: A centralised evaluation of 8088 patients from 10 study groups. Breast Cancer Res..

[4] Shui R., Yu B., Bi R., Yang F., Yang W. (2015). An interobserver reproducibility analysis of Ki67 visual assessment in breast cancer. PLoS ONE.

[5] Rimm D.L., Leung S.C., McShane L.M., Bai Y., Bane A.L., Bartlett J.M., Bayani J., Chang M.C., Dean M., Denkert C. (2019). An international multicenter study to evaluate reproducibility of automated scoring for assessment of Ki67 in breast cancer. Mod. Pathol..

[6] Polley M.Y.C., Leung S.C., McShane L.M., Gao D., Hugh J.C., Mastropasqua M.G., Viale G., Zabaglo L.A., Penault-Llorca F., Bartlett J.M. (2013). An international Ki67 reproducibility study. J. Natl. Cancer Inst..

[7] Naylor P., Laé M., Reyal F., Walter T. (2018). Segmentation of nuclei in histopathology images by deep regression of the distance map. IEEE Trans. Med. Imaging.

[8] Kumar N., Verma R., Anand D., Zhou Y., Onder O.F., Tsougenis E., Chen H., Heng P.A., Li J., Hu Z. (2019). A multi-organ nucleus segmentation challenge. IEEE Trans. Med. Imaging.

[9] Graham S., Vu Q.D., Raza S.E.A., Azam A., Tsang Y.W., Kwak J.T., Rajpoot N. (2019). Hover-net: Simultaneous segmentation and classification of nuclei in multi-tissue histology images. Med. Image Anal..

[10] Gamper J., Alemi Koohbanani N., Benet K., Khuram A., Rajpoot N. (2019). Pannuke: An open pan-cancer histology dataset for nuclei instance segmentation and classification. European Congress on Digital Pathology.

[11] Pachitariu M., Rariden M., Stringer C. (2025). Cellpose-SAM: Superhuman generalization for cellular segmentation. bioRxiv.

[12] Rao A., Krithika M., Kini H., Jeevitha, Basthikodi M. An Ensemble Deep Learning Framework for Automated Ki-67 Scoring in Breast Cancer. Proceedings of the 2025 IEEE International Conference on Distributed Computing, VLSI, Electrical Circuits and Robotics (DISCOVER).

[13] Kurniawan E., Ekoputro J.W., Soraya F., Rachmadi R.F., Susilo I., Purnama I.K.E. Enhancing Accuracy in Ki-67 Scoring: A Correlation-Based Approach Using Automated Image Analysis. Proceedings of the 2025 International Seminar on Intelligent Technology and Its Applications (ISITIA).

[14] Liu Q., Zhao Z., Lou L., Li Y., Wang S. (2025). Multi scale deep learning quantifies Ki67 index in breast cancer histopathology images. Sci. Rep..

[15] Li L., Han D., Yu Y., Li J., Liu Y. (2022). Artificial intelligence-assisted interpretation of Ki-67 expression and repeatability in breast cancer. Diagn. Pathol..

[16] Konsti J., Lundin M., Joensuu H., Lehtimäki T., Sihto H., Holli K., Turpeenniemi-Hujanen T., Kataja V., Sailas L., Isola J. (2011). Development and evaluation of a virtual microscopy application for automated assessment of Ki-67 expression in breast cancer. BMC Clin. Pathol..

[17] Laurinavicius A., Plancoulaine B., Laurinaviciene A., Herlin P., Meskauskas R., Baltrusaityte I., Besusparis J., Dasevicius D., Elie N., Iqbal Y. (2014). A methodology to ensure and improve accuracy of Ki67 labelling index estimation by automated digital image analysis in breast cancer tissue. Breast Cancer Res..

[18] Klauschen F., Wienert S., Schmitt W.D., Loibl S., Gerber B., Blohmer J.U., Huober J., Rüdiger T., Erbstößer E., Mehta K. (2015). Standardized Ki67 diagnostics using automated scoring—Clinical validation in the GeparTrio breast cancer study. Clin. Cancer Res..

[19] Schmidt U., Weigert M., Broaddus C., Myers G. (2018). Cell detection with star-convex polygons. International Conference on Medical Image Computing and Computer-Assisted Intervention.

[20] Weigert M., Schmidt U. Nuclei Instance Segmentation and Classification in Histopathology Images with Stardist. Proceedings of the The IEEE International Symposium on Biomedical Imaging Challenges (ISBIC).

[21] Stardist Contributors (2025). StarDist: Object Detection with Star-Convex Shapes. GitHub Repository. https://github.com/stardist/stardist.

[22] Ahn H.J., Jung S.J., Kim T.H., Oh M.K., Yoon H.K. (2015). Differences in clinical outcomes between luminal A and B type breast cancers according to the St. Gallen Consensus 2013. J. Breast Cancer.

[23] Nielsen T.O., Leung S.C.Y., Rimm D.L., Dodson A., Acs B., Badve S., Denkert C., Ellis M.J., Fineberg S., Flowers M. (2021). Assessment of Ki67 in breast cancer: Updated recommendations from the international Ki67 in breast cancer working group. JNCI J. Natl. Cancer Inst..

[24] Chollet F. (2015). Keras. https://keras.io.

[25] Gudlaugsson E., Skaland I., Janssen E.A., Smaaland R., Shao Z., Malpica A., Voorhorst F., Baak J.P. (2012). Comparison of the effect of different techniques for measurement of Ki67 proliferation on reproducibility and prognosis prediction accuracy in breast cancer. Histopathology.

[26] Yamamoto S., Chishima T., Mastubara Y., Adachi S., Harada F., Toda Y., Arioka H., Hasegawa N., Kakuta Y., Sakamaki K. (2015). Variability in measuring the Ki-67 labeling index in patients with breast cancer. Clin. Breast Cancer.

[27] Yerushalmi R., Woods R., Ravdin P.M., Hayes M.M., Gelmon K.A. (2010). Ki67 in breast cancer: Prognostic and predictive potential. Lancet Oncol..

